# Non-linear relationships of cerebrospinal fluid biomarker levels with cognitive function: an observational study

**DOI:** 10.1186/alzrt64

**Published:** 2011-02-17

**Authors:** Jonathan H Williams, Gordon K Wilcock, Jeffrey Seeburger, Aimee Dallob, Omar Laterza, William Potter, A David Smith

**Affiliations:** 1 OPTIMA, University of Oxford, John Radcliffe Hospital, Headington, Oxford, OX3 9DU, UK; 2 Clinical Neuroscience and Ophthalmology, Merck Research Laboratories, 351 N. Sumneytown Pike, North Wales, PA 19454, USA; 3 Clinical Development Laboratory, Merck Research Laboratories, 126 East Lincoln Avenue, Rahway, NJ 07065, USA

## Abstract

**Introduction:**

Levels of cerebrospinal fluid (CSF) β-amyloid (Aβ) and Tau proteins change in Alzheimer's disease (AD). We tested if the relationships of these biomarkers with cognitive impairment are linear or non-linear.

**Methods:**

We assessed cognitive function and assayed CSF Aβ and Tau biomarkers in 95 non-demented volunteers and 97 AD patients. We then tested non-linearities in their inter-relations.

**Results:**

CSF biomarkers related to cognitive function in the non-demented range of cognition, but these relations were weak or absent in the patient range; Aβ_1-40_'s relationship was biphasic.

**Conclusions:**

Major biomarker changes precede clinical AD and index cognitive impairment in AD poorly, if at all.

## Introduction

The incidence and prevalence of Alzheimer's disease (AD) double every five years from age 65, to affect over one quarter of people aged over 85 [1,2]. AD pathology can develop long before clinical symptoms [3-5]. This means that, ideally, disease-modifying treatments should begin before diagnosis [6]. Consequently, there is much interest in finding biomarkers that can predict the onset of AD [7]. There is also much interest in finding biomarkers to assess treatment effects [8]. Leading candidates for these roles are β-amyloid (Aβ) and Tau proteins in the cerebrospinal fluid (CSF) [9-15]. To date, nearly all studies of CSF biomarkers have related them to diagnostic categories -AD and mild cognitive impairment (MCI). However, the boundaries of diagnostic categories, particularly MCI, are uncertain [16-18]. We, therefore, related CSF biomarkers directly to cognitive test scores.

The form of the relationship between biomarker levels and cognitive scores is important. To predict the onset of AD, a biomarker should relate to cognitive decline pre-clinically. Conversely, to monitor disease progression and treatment response, a biomarker should relate to cognitive level in the range of clinical dementia. These relationships may be such that alterations in biomarker expression may precede, coincide with, or lag behind changes in cognitive status. The simplest assumption would be that the relation of CSF amyloid and Tau biomarkers with cognitive function is linear from cognitive normality through MCI to AD. If so, then these biomarkers might be useful for both the prediction of AD and monitoring its progression. However, most studies that related CSF Aβ and Tau levels to cognitive scores [9,19,20] did not test this assumption of a linear relationship and one study [21] found no relationship in AD patients. Moreover, recent studies that used diagnostic categories have provided evidence of biphasic changes in levels of putative biomarkers between controls, MCI and AD patients in CSF [21,22] and blood [23,24]. These findings support Combrinck's hypothesis [25] that biomarkers may relate non-linearly to cognitive function. Here, we test this hypothesis further by analysing the forms of the relations between CSF biomarkers and cognitive scores.

We have previously reported a biphasic relation between CSF PGE_2 _levels and cognitive scores, using an analysis of covariance with polynomial trends [25]. The present study uses change-point analyses with highly robust linear regression to test for non-linearity in cross-sectional relations between cognitive scores and CSF Aβ and Tau moieties.

## Materials and methods

### Participants

Participants were volunteers in the Oxford Project To Investigate Memory and Ageing (OPTIMA), a naturalistic longitudinal study of memory and ageing. All OPTIMA's protocols received prior ethical approval from the local research ethics committee (COREC #1656). OPTIMA is a convenience sample of patients with dementia and non-demented volunteers of similar age. We have described OPTIMA's recruitment and assessment protocol previously [26]. Briefly, at their initial assessment all participants underwent a physical examination, blood tests, CT scan and cognitive assessment using the Cambridge Cognitive examination (CAMCOG) [27]. We also obtained a detailed history from participants and an informant. We invited participants who could give valid consent to undergo a lumbar puncture (LP).

Clinical diagnoses of Alzheimer's disease or Other Dementia Syndromes used the National Institute of Neurological and Communicative Disorders and Stroke (NINCDS) criteria [28]. OPTIMA has a high rate of autopsy acceptance (over 80%). Details of OPTIMA's neuropathological examinations are available elsewhere [29]. We supplanted clinical diagnoses whenever possible by neuropathological diagnoses using Consortium to Establish a Registry for Alzheimer's Disease (CERAD) criteria [30]. The present analysis did not include consideration of Braak staging [3] in the determination of neuropathological diagnosis or its relationship to the variables studied.

The present report includes data from all non-demented controls and AD patients aged over 60 who underwent lumbar puncture and whose CAMCOG data were complete. It excludes patients with clinical or neuropathological diagnoses of non-Alzheimer dementias.

### Lumbar punctures

All LPs used standard clinical techniques [31]. Most took place in the late morning. LP has a low risk of adverse effects in our cohort [31]. We collected the CSF samples into polystyrene tubes.

### CSF assays

We centrifuged CSF samples for 10 minutes at 4°C at 1,000 g to remove cells and stored the supernatant in aliquots of 0.5 ml in polypropylene tubes at -70°C. We assayed levels of CSF Aβ and Tau moieties using commercial kits. No sample underwent any freeze-thaw cycles between collection and these assays.

#### Aβ_1-40 _assay

Aβ_1-40 _was measured in the CSF with a human Aβ_1-40 _Colorimetric solid phase sandwich Enzyme Linked Immuno-Sorbent Assay (ELISA) kit (catalogue # KHB3482, BioSource International, Camarillo, CA, USA) following the manufacturer's recommendations. This assay employs a mouse monoclonal antibody specific for the N-terminal half of Aβ_1-40 _as capture and a rabbit anti- Aβ_1-40 _neo-epitope (secondary antibody). The detection antibody consisted of a secondary anti-rabbit IgG:horse radish peroxidase (HRP) conjugate. HRP catalyzes the formation of a chromophore, tetramethylbenzidine (TMB), which was measured at 450 nm. A total of 100 μL of the sample (CSF diluted 1:20 in assay buffer) was used in this assay. The standards were provided in the BioSource assay kit and they ranged from 15.6 to 1000 pg/mL.

#### Aβ_1-42 _assay

Aβ_1-42 _was measured with Innotest™ Aβ_1-42 _ELISA kit (Innogenetics Inc., Cat. #80040, Ghent, Belgium) following the manufacturer's recommendations with some modifications. Aβ_1-42 _present in human CSF samples was first captured with a mouse monoclonal antibody specific for the C-terminal half of Aβ The detection system employs an N-terminal specific biotinylated mouse monoclonal antibody and a secondary conjugate made of HRP labeled strepavidin. The HRP is used to convert tetramethyl benzidine to a chromophore which is quantitatively measured at 450 nm. A total of 100 μL of the sample (CSF Diluted 1:3 with Sample Diluent) was used in each reaction. Aβ_1-42 _standard was purchased from American Peptide (Sunnyvale, CA, USA) and the concentration was determined by amino-acid analysis. Standard concentrations in the assay ranged from 5.45 to 350 pg/mL.

#### Tau assay

Total Tau (t-Tau) expression was measured with a human Tau (hTAU AG Innotest™) ELISA kit (Innogenetics Inc., catalogue number 80226, Ghent, Belgium) following the manufacturer's recommendations. The analyte was first captured with a monoclonal antibody specific for all isoforms of Tau, and then subsequently bound by two biotinylated Tau-specific antibodies. The final detection was performed by peroxidase-labeled streptavidin. A total of 25 μL of the sample was tested undiluted. The standards were supplied with the kit and ranged from 37.5 to 1200 pg/mL.

#### Phospho-Tau assay

Phosphorylated Tau-181 (pTau-181) was measured with the Phospho-TAU (_181P_) Innotest™ ELISA kit (Innogenetics Inc., catalogue number 80062, Ghent, Belgium), following the manufacturer's recommendations. The analyte was first captured with an antibody specific for all isoforms of Tau and then detected with a second detection antibody which specifically detects Tau molecules phosphorylated at threonine 181 (phospho-tau-181). A 75 μL sample was tested undiluted. The standards were supplied with the kit and ranged from 15.6 to 500 pg/mL.

#### Assay validation

All assays were analytically validated (inter- and intra-assay precision, freeze/thaw stability, linearity, spike recovery, and sensitivity). In addition, quality control samples (low, medium, and high) were run on all plates and were used as part of the run acceptance criteria. All analytes were found to be stable (<20% change) after three freeze-thaw cycles. All sample analyses were performed in duplicate.

For the Aβ_1-40 _assay, the intra- and inter-assay percent coefficient of variation (% CV) ranged from 4.1% to 7.6% and 9.4% to 12.5%, respectively. The spike recovery was determined to be 105 to 114% and the lower limit of reliable quantitation was 17.8 pg/mL.

For the Aβ_1-42 _assay, the intra- and inter-assay % CV ranged from 3.7 to 4.7% and 5.9 to 7.6%, respectively. The spike recovery was determined to be 70 to 109% and the lower limit of reliable quantitation was 24.5 pg/mL.

For the t-Tau assay, the intra- and inter-assay % CV ranged from 3.8 to 9.0% and 6.2 to 7.2%, respectively. The spike recovery was determined to be 105 to 116% and the lower limit of detection was 37.5 pg/mL.

For the p-Tau assay, the intra- and inter-assay % CV ranged from 1.3 to 2.2% and from 4.9 to 5.3%, respectively. The spike recovery was determined to be 84 to 89% and the lower limit of detection was 15.6 pg/mL.

### Statistics

All statistical analyses used the open-source statistical programming language 'R' [32]. We used the Wilcoxon-Mann-Whitney signed rank test and Pearson's χ^2 ^to compare the demographic characteristics of the patient and non-demented groups.

We first performed omnibus tests for non-linearity using multivariate analysis of variance (MANOVA) [33]. The first MANOVA tested the dependence of all four biomarkers on age, gender, storage time, CAMCOG score and assay group. A second MANOVA included all the above variables and a second CAMCOG term representing an inflection in the relationship with cognitive function, corresponding with our previous work [25]. That study showed an inflection in the relation between CSF PGE_2 _levels and CAMCOG learning sub-scale scores at a score of 11, the lowest limit of the non-demented range. In the present study, we first ascertained the total CAMCOG score that corresponded best with a CAMCOG learning sub-scale score of 11, then programmed an inflection at that total score (89).

The strategy of pre-defining an inflection point for all CSF biomarkers may be sub-optimal if different biomarkers show different inflection points. Therefore, having obtained evidence of a significant inflection in our MANOVA (see Results), we tested for change-points in the relations between each individual biomarker level and CAMCOG score. These change-point analyses were of two kinds. First, we tested for changes in the overall level of each biomarker in relation to CAMCOG. These analyses used the "Fstats" and "breakpoints" procedures for testing structural changes in linear regression models [34]. Second, we tested for change-points in the slope of the relation between each biomarker and CAMCOG. These analyses used the "linearSegmentation" procedure for piecewise linear segmentation of a time series [35]. This procedure requires data in a continuous equally-spaced series with one observation at each point. We converted the CAMCOG scores to a series of this kind by ranking them and randomly splitting ties. Since the order of the random splits may affect the result of the change-point analysis, we repeated the analysis 1,000 times with a different random seed to split ties in each repetition. Each analysis progressively increased the window size and tolerance angle in each repetition, until the procedure generated a single change-point for each biomarker. We recorded the change-points for each biomarker in each of the 1000 repetitions. Finally, we extracted the median of these 1000 estimates. Hence, for each biomarker we derived two change-points: 1) CAMCOG scores at which the biomarker changed its level, and 2) change in the slope of the relation between each biomarker and CAMCOG. These two change-points were very similar for each biomarker, so we used their mean in further analyses.

**Figure 1:**
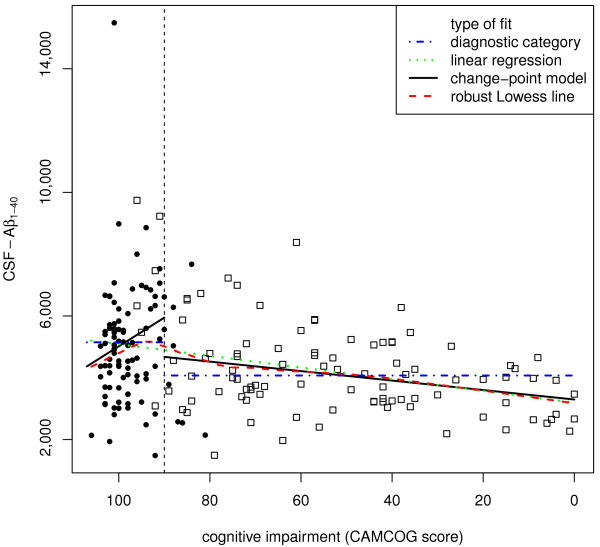
**The dependence of CSF Aβ_1-40 _levels (y-axis) on CAMCOG score (x-axis), which decreases from left to right, as cognitive impairment increases**. The Figure represents the fits of four different models to the data. (1) The horizontal blue dot-dash lines represent the means for each diagnostic category. (2) The straight green dotted line represents a robust linear regression of CSF Aβ_1-40 _levels on CAMCOG score that does not include the change point. (3) The solid black lines represent the robust regression model that takes into account the change point (vertical dashed line). (4) The curvilinear dashed red line is the model-free robust locally-fitted Lowess line. Closed circles represent cognitively healthy participants and open squares represent patients with AD.

**Figure 2:**
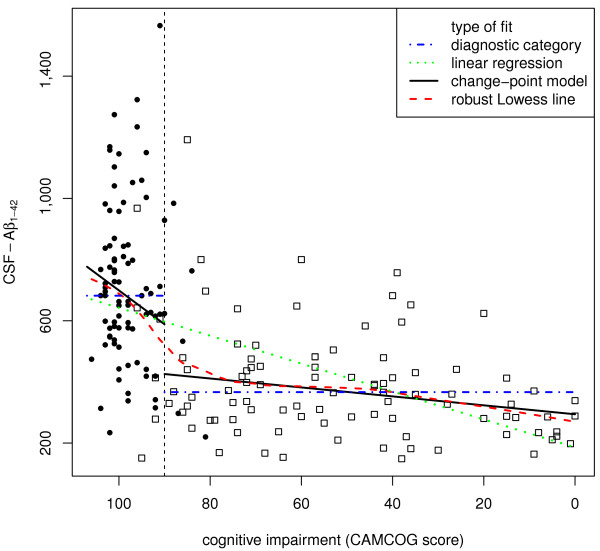
**The dependence of CSF Aβ_1-42 _levels on CAMCOG score**. All details as for Figure 1.

**Figure 3:**
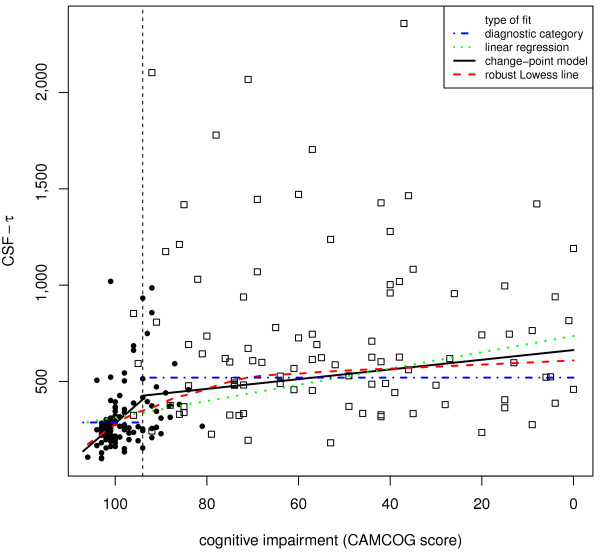
**The dependence of CSF Tau levels on CAMCOG score**. All details as for Figure 1.

**Figure 4:**
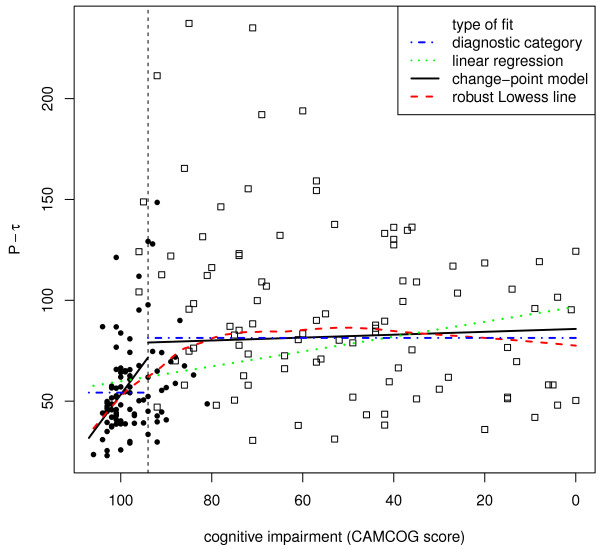
**The dependence of CSF phospho-Tau levels on CAMCOG score**. All details as for Figure 1.

We assessed the validity of the change-point for each biomarker in three ways. First, we visually compared the change-point and model-free robust Lowess fits (Figures 1, 2, 3, 4). Second, we compared the variances and medians of each biomarker on each side of its change-point using simple variance ratios and Wilcoxon-Mann-Whitney (WMW) tests. Third, we compared the relationship of the biomarker with CAMCOG on each side of its change-point using Spearman's ρ and robust linear modelling (rlm) [36]. The rlms used highly robust M-estimation (via the 'MM' option) with a breakdown point of 0.5 and 95% relative efficiency at the normal.

## Results

### Participants

A total of 192 participants fulfilled the inclusion criteria; 97 received a diagnosis of AD and 25 of the non-demented volunteers received a designation of MCI. We obtained post mortem neuropathological confirmation of the diagnosis in 90% of the AD patients (88/97) and a third of the non-demented goup (30/95). All participants were white West Europeans. Table 1 shows their demographic and clinical characteristics. The age and gender distributions of the non-demented participants and AD patients did not differ (age: WMW *P *= 0.11; gender: Fisher exact *P *= 0.15). Compared with non-demented participants, AD patients had lower MMSE and CAMCOG scores and shorter follow-up (all WMW *P-*values < 0.001). We have provided further clinical details in Additional file 1. The role of the diagnostic procedures in the present study was to ensure that the study group did not include participants with non-Alzheimer dementias. Therefore, neither clinical nor neuropathological diagnostic categorisations played any part in the design or interpretation of our change-point analyses, which concern only the forms of the relations between CSF biomarkers and CAMCOG scores.

**Table 1:** Demographic and clinical characteristics of the participants

	Gender	Age	MMSE	CAMCOG	Follow-up (years)
Non-demented	48F: 47M	72.0 (66.0-78.8)	29 (28-30)	99 (81-106)	8.3 (4.7 12.9)
Alzheimer	60F: 37M	74.6 (69.7 79.8)	15 (9 23)	57 (10 96)	3.6 (1.4 6.2)

The values for Age, MMSE, CAMCOG and Follow-up are medians and inter-quartile ranges.

### Multivariate inflection model

The inflection point in the total CAMCOG score corresponding to our previous finding [an inflection at a CAMCOG learning sub-scale score of 11 - see 25] was 89. Biomarker levels showed different levels and their relations with CAMCOG scores showed different slopes above and below total scores of 89 (levels: F = 11.5, 4/167 df, *P *< 0.0001. Slopes: F = 3.31, 4/167 df, *P *= 0.012) (see Table 2 and Figures 1, 2, 3, 4).

**Table 2:** Parameters of the CSF biomarkers and their relations to cognitive impairment, above and below their change-points

CSF Biomarker	Aβ_1-40_		Aβ_1-42_		Tau		Phospho-Tau	
Cf change-point	Above	Below	Above	Below	Above	Below	Above	Below
Median	5,052	3,937**	682	350**	259	568**	50	80**
Variance	3,857,482	1,854,983**	36,846	71,482**	176,946	73,418**	1,700	1,000**
Spearman's ρ	0.21*	-0.23*	-0.24*	-0.12	0.34**	0.28**	0.32**	0.16
Robust slope	93**	-15**	-0.048**	-0.002	0.049*	0.004**	0.041**	0.004**

The values in the first row are the raw biomarker levels (pg/mL); the second row shows the variance of the median-standardised levels; the third row shows Spearman's ρs for the rank correlations of biomarker levels with CAMCOG scores; the fourth row shows the slopes from the robust regressions of median-standardised biomarker levels. For each biomarker, the "Above" and "Below" columns shows values for CAMCOG scores above and below the change-point, respectively. Note that the correlations and slopes take cognitive impairment as the x-axis, which is the reverse of CAMCOG scores. Key:- ^(^*^)^: 0.10 >*P *> 0.05; *: *P *< 0.01; **: *P *< 0.01. For medians and variances, probabilities refer to the comparison of values above and below the change-point; for Spearman's ρ and slopes they refer to comparison of values with zero.

### *CSF Aβ_1-40_*

CSF Aβ_1-40 _levels showed biphasic dependence on CAMCOG scores, with the change-point at a CAMCOG of 90 (Figure 1). The change-point model fitted the data better than the simpler linear model (F = 3.27, 2/187df, *P *= 0.04). There was a positive relation of Aβ_1-40 _with CAMCOG scores above the change point (rlm: t = 2.65, 187 df, *P *= 0.009) but a negative relation below it (rlm: t = -3.25, 187 df, *P *= 0.001; Table 2). Congruent with this, the Spearman's ρs on each side of the change-point were opposite in sign and both differed from zero (Table 2; Figure 1). The median and variance of Aβ_1-40 _levels were greater above the change-point than below it (median: WMW *P *< 0.001; variance: F = 1.95; 96/96 df; *P *< 0.0001; see Table 2). In summary, the levels and variability of CSF Aβ_1 to 0 _and the sign of its relation with CAMCOG differed above and below the change-point.

### *CSF Aβ_1-42_*

CSF Aβ_1-42 _levels showed monotonic but non-linear dependence on CAMCOG scores with the change-point at a CAMCOG of 90 (Figure 1b). The change-point model fitted better than the simpler linear model (F = 11.2, 2/178 df, *P *< 0.001). There was a marked negative relation of Aβ_1-42 _with CAMCOG scores above the change point (rlm: t = -4.29, 178 df, *P *< 0.001), but no relation below it (rlm: t = -1.17, 178 df, P = 0.24) (Table 2; Figure 2). Congruent with this, Spearman's ρs showed a negative relation only with CAMCOG scores above the change point (see Table 2). The median and variance of Aβ_1-42 _levels above the change-point were greater than below it (median: WMW *P *< 0.001; variance: F = 2.11; 92/100 df; *P *< 0.001). In summary, the levels and variability of CSF Aβ_1-42 _and the slope of its relation with CAMCOG differed above and below the change-point.

### CSF Tau

CSF Tau levels showed monotonic but non-linear dependence on CAMCOG scores with the change point at a CAMCOG of 94. The change-point model fitted better than the simple linear model (F = 5.39, 2/187 df, *P *= 0.005). Tau levels related to CAMCOG scores more strongly above the change-point (rlm: t = 2.01, 187 df, *P *= 0.046) than below it (t = 4.46, 187 df, *P *< 0.001) (Table 2; Figure 3). Spearman's ρs showed positive relations with cognitive impairment both above and below the change point (see Table 2). The median and variance of CSF Tau levels in the CAMCOG range below the change-point were much greater than above it (median: WMW *P *< 0.001; variance: F = 7.06; 119/73 df; *P *< 0.001). In summary, the levels and variability of CSF Tau and the slope of its relation with CAMCOG differed above and below the change-point.

### CSF phospho-Tau

CSF phospho-Tau levels showed monotonic but non-linear dependence on CAMCOG scores, with the change point at a CAMCOG of 93. The change-point model fitted better than the simple linear model (F = 10.2, 2/187 df, *P *< 0.001). phospho-Tau levels related directly to CAMCOG scores above the change-point (t = 2.39, 187 df, *P *= 0.018), and below it (t = 2.48, 187 df, *P *= 0.014) (Table 2; Figure 4). The Spearman's ρs showed a positive relation with cognitive impairment only in the CAMCOG range above the change point (see Table 2). The median and variance of CSF phospho-Tau levels in the CAMCOG range below the change-point were greater than above it (median: WMW *P *< 0.001; variance: F = 2.82; 113/79 df; *P *< 0.001). In summary, the levels and variability of CSF phospho-Tau and the slope of its relation with CAMCOG differed above and below the change-point.

## Discussion

CSF β-amyloid and Tau peptides showed distinctive non-linear relationships with cognitive scores. These relationships were strongest in the "normal" CAMCOG range and every biomarker's change-point was near the lower boundary of this range. These non-linearities are inconsistent with a simple linear relationship between central amyloid and Tau levels and cognitive impairment which underlies the view that modulating central amyloid and Tau may prevent cognitive decline in dementia [37,38]. Instead, they suggest that most changes in central amyloid and Tau precede the development of clinical dementia [39].

### Reliability of our findings

The findings of our change-point analyses are consistent with published data. First, our re-analysis of data shown in Figure 2 in Maruyama [19] shows a change-point in the relationship between MMSE scores and CSF Aβ_1-42_. This resembles our present finding (see our Figure 2) in that there is no relation between MMSE and Aβ_1-42 _across MMSE scores below 20 (t = 0.21, NS), but a significant relation across MMSE scores above 20 (t = 2.08, 76 df, *P *= 0.041). Second, our findings that relations between CSF biomarkers and CAMCOG were weak or absent below the change-points parallel the observations of Vemuri [40] in AD patients; the stronger relations that we found above the change-points may also parallel Vemuri's report [40] that biomarker levels differed in their Controls and people with MCI. Third, our finding of an inverted-U relation between Aβ_1-40 _and CAMCOG scores parallels the report that Aβ_1-40 _levels were high in MCI patients who progressed to AD [21]. Additional factors that strengthen our change-point results are (a) the strong resemblance between the change-point-based robust regression models and the model-free Lowess fits (see Figures 1, 2, 3, 4); (b) the similarity of the change-points (in absolute CAMCOG scores) over all four biomarkers, despite the different forms of each biomarker's relation with cognitive function; (c) the significant differences between the variability and overall levels of the biomarkers on each side of the change-points. Overall, therefore, the change-points we describe are unlikely to be artefactual and relations between CSF biomarkers and cognition are probably non-linear.

The change-points that we defined are near the bottom of the range of CAMCOG scores in our non-demented volunteers. Hence, our findings are consistent with many previous reports of higher levels of CSF Tau moieties and lower levels of CSF amyloid moieties in AD [22,25,41,42]. However, we prefer our change-point categories to the diagnostic classification because they derive from a model-free method, whereas the definition of Alzheimer's disease can vary [43,44]. Moreover, our results indicate that neither the diagnostic classification, nor the diagnostically agnostic linear regression fully describes the relations between cognitive function and CSF biomarkers. Figures 1, 2, 3, 4 each show the four models that assume that the relations between CSF biomarker levels and cognitive function are either (a) simply linear (green dotted lines) or (b) simply categorical (blue dot-dash lines). In each case these simple relations fit the data quite well, but the non-linear relations based on the change-point analyses (c - black solid lines) fit better and more closely resemble the model-free robust Lowess fits (d - red dashed lines). These improvements in fit are not large, but their theoretical importance outweighs their actual size, as we discuss below.

### *CSF Aβ_1-40_*

CSF Aβ_1-40 _showed biphasic dependence on cognitive scores (Figure 1), with maximal levels near the lower limit of our non-demented participants' CAMCOG scores. This is consistent with the findings that MCI patients who progressed to AD had high CSF Aβ_1-40 _[21] and CSF BACE activity is higher in MCI than in either controls or AD patients [22]. It also fits with findings of biphasic changes in plasma amyloid levels [24]. Zhong *et al. *[22] interpreted their findings as supporting the amyloid cascade hypothesis. However, the biphasic relationship of Aβ_1-40 _admits at least two other overlapping interpretations. First, it is consistent with Combrinck's hypothesis [25] that a pathogenic mechanism may 'burn out' early in AD. Second, it is consistent with the hypothesis that Aβ may be protective [45,46], and AD occurs only when pathogenic mechanisms overwhelm the amyloid response. Further studies are necessary to explore these three possibilities.

### CSF Aβ_1-42_

CSF Aβ_1-42 _showed an overall inverse relationship with the degree of cognitive impairment (Figure 2). This fits with many reports of low Aβ_1-42 _in AD patients [40-42]. Our findings extend those reports by showing that Aβ_1-42 _depends on cognitive level only in the range of non-demented volunteers' CAMCOG scores, not in the patients' range. The absence of any relation between CSF Aβ_1-42 _and CAMCOG scores below the change-point (in the patient range) mirrors the recent report of Vemuri *et al. *[40]. Together, these results indicate that low Aβ_1-42 _may provide an early marker of likely progression to AD, rather than an index of the severity of pathology in established AD. The contrast between the monotonic relation of Aβ_1-42 _with cognitive level and the biphasic relation of Aβ_1-40 _is striking (compare Figures 1 and 2). This contrast fits with observations that a low ratio of Aβ_1-42_/Aβ_1-40 _associates with imminent risk of mild cognitive impairment and Alzheimer's disease [47-49]. Together, these findings highlight a need for further studies to explain why the ratio of Aβ_1-42_/Aβ_1-40 _may vary [50].

### CSF Tau and phospho-Tau

We found high CSF Tau moieties in AD patients [51]. Our results extend earlier reports by showing that, like Aβ_1-42_, Tau and phospho-Tau levels relate to CAMCOG mainly above the change-point (in the non-demented range of scores). Again, paralleling Vemuri *et al.*'s report [40], we found no simple relation between CSF phospho-Tau and CAMCOG scores below the change-point, in the patient range (Figures 3, 4). Together, these results reinforce the view that phospho-Tau may provide an early marker of likely progression to AD, but not an index of the severity of pathology in established AD. The findings that Aβ_1-42 _and phospho-Tau show opposite relations with cognitive scores within the non-demented range fits with reports that the ratio of Aβ_1-42_/Tau may be a sensitive indicator of progression to AD [41,52,53].

### Heteroscedasticity and measurement technicalities

The variances of the CSF biomarkers differed above and below their change-points. The simplest explanation of this is that the variance related to the mean, as often occurs in biological variables. It may also represent individual differences [54], or variations in pathogenic processes or cognitive profiles [55]. For example, the heteroscedasticity may possibly relate to ApoE status. We did not test relations of ApoE alleles with change-points, since we had no *a priori *hypothesis about this. Further studies should address this potentially important possibility. Whatever its explanation, the heteroscedasticity in every biomarker indicates a need for caution when interpreting biomarker levels in patient and control groups, or in individuals [56]. For now, we note that our use of highly robust linear modelling with a breakdown point of 0.5 [36] guards against potential bias in our analyses due to heteroscedasticity.

We collected CSF samples in polystyrene tubes and stored them in polypropylene tubes. Both kinds of tube can adsorb large amounts of biomarker molecules [57,58]. If such adsorption saturates asymptotically, and so depends non-linearly on initial biomarker concentration, then this might possibly contribute to both the heteroscedasticity and the non-linear relations between biomarker levels and cognitive function that we found. However, we think this unlikely, as follows. If adsorption of biomarkers tends to cause floor effects in their apparent levels, then cognitive function might relate to biomarker levels only when they exceed this artefactual floor. Such floor effects could possibly explain our finding that CAMCOG scores did not relate to Aβ_1-42 _in the patient range of CAMCOG scores (because Aβ_1-42 _levels are lowest in this range and so most susceptible to adsorption-mediated floor effects). However, it cannot simultaneously explain the inverted-U biphasic relation of CAMCOG with Aβ_1-40_; nor can it explain the direct relationships of the CAMCOG scores with tau and p-tau in the non-demented range of CAMCOG scores (where lower Tau/p-Tau levels are potentially more susceptible to adsorption-mediated floor effects). Even for Aβ_1-42_, this account appears tenuous, in view of Bjerke's observation that detergent treatment released similar percentages of Aβ_1-42 _from adsorption in both control and patient samples [57]. In summary, we think it unlikely that measurement technicalities can account for the non-linear dependence of CSF biomarkers on cognitive function that we observed.

We related CSF biomarker levels to raw CAMCOG scores. Hence, apparent non-linearities in the relationships may in part reflect non-linearities in the metric properties of the CAMCOG (for example [59]). In particular, the narrow range of CAMCOG scores above the change-points may contribute to the differences in the slopes of their relations with biomarkers, since CAMCOG scores here may relate less strongly to true cognitive ability. However, this cannot account for (a) the inverted-U relation of Aβ_1-40 _with CAMCOG scores; nor for (b) the differences in Spearman's rank correlation coefficients (which is independent of the metric) for biomarkers and CAMCOG scores above and below the change-points; nor for (c) the differences in variance of biomarkers above and below the change-points. Therefore, while the slopes of the relationships that we found on each side of the change-points may vary under non-linear transformations of the CAMCOG scores, it seems unlikely that our use of raw CAMCOG scores can account for the existence of the change-points.

### Use of CAMCOG scores as metric for dementia

The main limitation of our change-point analyses is that they used only cross-sectional data. Their use of CAMCOG scores as the metric for dementia removes time from the analysis of progression. The absolute cognitive level is clinically meaningful regardless of age or the duration of symptoms. Therefore, using it as the metric for progression of pathological mechanisms may be preferable to using time. We know that individuals' CAMCOG scores can decline over time through the normal and patient ranges [59]. Hence, it is tempting to view the cross-sectional dependence of CSF biomarkers on CAMCOG scores as a model of an individual's likely progression over time. However, the heteroscedasticity that we observed (see above) means that individuals might show important variations from this model. Consequently, it would be inappropriate at this stage to conclude that the non-linear cross-sectional relationships that we observed can indicate which non-demented people will progress to AD, or which AD patients will decline faster. Conversely, the cognitive stability of many non-demented participants implies that the cross-sectional relations of CSF biomarkers with their cognitive function may index long-term adaptations to factors that pre-dispose to AD [60]. Alternatively, these cross-sectional relationships may reflect Vemuri *et al.'*s [40] observation that levels of biomarkers differed between controls and MCI groups, since we did not distinguish these. MCI may be stable, remit, or progress to AD [17,18]. Hence, the cross-sectional relations of biomarkers with CAMCOG scores in the non-demented range may reflect a mix of long-term adaptations and of vulnerabilities to progression. Further longitudinal studies relating CSF biomarker levels to cognitive function are necessary to define more precisely the links between CSF biomarkers and the putative primary pathogenic processes of AD.

### Generalizabilty

The generalizabilty of our study may be comparable with other reports of CSF biomarkers. Participants in all such studies are partly self-selected, both for entry to the cohort and for consenting to LP. OPTIMA is a convenience cohort from a relatively small geographical area in and around Oxford. Our study group was relatively homogeneous and most non-demented volunteers were cognitively stable, with few converting to AD, despite our long follow-up. Together, these two considerations indicate that our study group is unlikely to be representative of the general population. Even so, the consistency of our results with previous reports, both with regard to differences in CSF biomarkers between patients and non-demented controls and to non-linearity [cf. [21-24]], even in a Japanese sample [19], implies that our findings may reflect general phenomena. Two further aspects of our study may improve its generalizability. First, we confirmed the diagnosis of AD and excluded non-Alzheimer dementias via neuropathological examination in most patients (though people who consent to autopsy are non-representative of the general population [61]). Second, we related CSF biomarker levels directly to cognitive scores. Neuropathological designations and cognitive test scores may provide a firmer basis for generalization than clinical diagnoses, whose boundaries are uncertain [16-18,43,44]. Overall, then, our study compares favourably with other reports in this field.

## Conclusions

The change-points in relation between cognitive function and all biomarkers were in the "normal" range of CAMCOG scores. This is consistent with increasing evidence that many non-demented older people have some AD pathology [3-5], which implies that disease-modifying treatments may be maximally effective for prophylaxis before clinical dementia occurs [6,62]. Hence our results could help explain recent reports that anti-amyloid treatments, such as tarenflurbil and anti-amyloid immunotherapy, had no effect in established AD [63-65], assuming that these treatments had anti-amyloid effects at the doses tested. The biphasic relationship we observed for Aβ_1-40 _is also consistent with the possibility that it may contribute to neuronal protection or maintenance [45,60,66,67]. If this were ultimately to prove to be the case, then BACE inhibition at critical stages might be detrimental. Whereas the field focuses most on Aβ_1-42_, this possibility suggests that more attention should be given to understanding the physiological role(s) of Aβ_1-40_.

## Abbreviations

% CV: coefficient of variation; AD: Alzheimer's disease; Aβ: beta amyloid; BACE: beta amyloid precursor protein cleaving enzyme; CAMCOG: Cambridge Cognitive examination; CERAD: Consortium to Establish a Registry for Alzheimer's Disease; COREC: Central Oxford Research Ethics Committee; CSF: Cerebrospinal fluid; CT: computerised tomography; ELISA: enzyme-linked immunosorbent assay; HRP: horse radish peroxidise; IgG: immunoglobulin-G; LP: lumbar puncture; MANOVA: multivariate analysis of variance; MCI: Mild Cognitive Impairment; MMSE: Mini Mental State Examination; NINCDS: National Institute of Neurological and Communicative Disorders and Stroke; NS: non-significant; OPTIMA: Oxford Project To Investigate Memory and Ageing; PGE_2_: prostaglandin E_2_; p-tau: phosphorylated tau; rlm, robust linear model; TMB: tetramethylbenzidine; WMW: Wilcoxon-Mann-Whitney.

## Competing interests

JS, AD, OL, and WP are employees of and own stock in Merck Sharp & Dohme Corp. and GKW is co-Editor-in-Chief of *Alzheimer's Research & Therapy*. The other authors declare that they have no competing interests.

## Authors' contributions

JHW conceived and performed the modelling and analysis. JS, AD, OL and WP were responsible for the biomarker studies. All authors contributed to the interpretation of the results and drafting of the final report.

## Supplementary Material

Additional file 1 **Supplementary methods**. A document outlining additional diagnostic considerations. Click here for file
