# Supplementary material for: Non-linear relationships of cerebrospinal fluid biomarker levels with cognitive function: an observational study
Source: Alzheimers Res Ther. 2011 Feb 17;3(1):5. doi: 10.1186/alzrt64 (PMC3109414; doi:10.1186/alzrt64)
Supplement: Additional file 1 — Supplementary methods. A document outlining additional diagnostic considerations. [file alzrt64-S1.DOCX]

**Online supplement**

We supplanted clinical diagnoses by neuropathological diagnoses whenever possible. Since we excluded non-Alzheimer dementias, this means that the CAMCOG scores principally reflect the severity of Alzheimer pathology (though not exclusively – see below).

25 participants received clinical diagnoses of MCI. Additionally, the figures show that a dozen participants whom we classified as 'patients' had initial CAMCOG scores in the non-demented ‘control’ range. Therefore, the proportion of non-demented 'controls' with MCI was 26% of whom half have converted to AD. This latter value does not include 10 other control volunteers whom we did not classify as MCI, but who showed declines of CAMCOG scores. Two showed very low CAMCOG scores in the context of physical illness: the decline in one was terminal and there was no autopsy evidence of Alzheimer disease; the other's CAMCOG scores rebounded to the normal range. Eight further controls showed progressive cognitive impairment to CAMCOG scores less than 80, more than 5 years after their LP assessment. These declines may also relate to physical illness and disability and none of the eight has a diagnosis of Alzheimer’s disease: three have undergone autopsy and all three received the CERAD designation “Normal brain”. These diagnostic considerations have no part in our change-point analyses.
